# Metabolomics Profiling As a Diagnostic Tool in Severe Traumatic Brain Injury

**DOI:** 10.3389/fneur.2017.00398

**Published:** 2017-08-18

**Authors:** Jussi P. Posti, Alex M. Dickens, Matej Orešič, Tuulia Hyötyläinen, Olli Tenovuo

**Affiliations:** ^1^Division of Clinical Neurosciences, Department of Neurosurgery, Turku University Hospital, Turku, Finland; ^2^Division of Clinical Neurosciences, Department of Rehabilitation and Brain Trauma, Turku University Hospital, Turku, Finland; ^3^Department of Neurology, University of Turku, Turku, Finland; ^4^Turku Centre for Biotechnology, University of Turku, Turku, Finland; ^5^Department of Chemistry, Örebro University, Örebro, Sweden

**Keywords:** traumatic brain injury, metabolomics, biomarker, neuromonitoring, outcome, mass spectrometry

## Abstract

Traumatic brain injury (TBI) is a complex disease with a multifaceted pathophysiology. Impairment of energy metabolism is a key component of secondary insults. This phenomenon is a consequence of multiple potential mechanisms including diffusion hypoxia, mitochondrial failure, and increased energy needs due to systemic trauma responses, seizures, or spreading depolarization. The degree of disturbance in brain metabolism is affected by treatment interventions and reflected in clinical patient outcome. Hence, monitoring of these secondary events in peripheral blood will provide a window into the pathophysiological course of severe TBI. New methods for assessing perturbation of brain metabolism are needed in order to monitor on-going pathophysiological processes and thus facilitate targeted interventions and predict outcome. Circulating metabolites in peripheral blood may serve as sensitive markers of pathological processes in TBI. The levels of these small molecules in blood are less dependent on the integrity of the blood–brain barrier as compared to protein biomarkers. We have recently characterized a specific metabolic profile in serum that is associated with both initial severity and patient outcome of TBI. We found that two medium-chain fatty acids, octanoic and decanoic acids, as well as several sugar derivatives are significantly associated with the severity of TBI. The top ranking peripheral blood metabolites were also highly correlated with their levels in cerebral microdialyzates. Based on the metabolite profile upon admission, we have been able to develop a model that accurately predicts patient outcome. Moreover, metabolomics profiling improved the performance of the well-established clinical prognostication model. In this review, we discuss metabolomics profiling in patients with severe TBI. We present arguments in support of the need for further development and validation of circulating biomarkers of cerebral metabolism and for their use in assessing patients with severe TBI.

## Introduction

Severe traumatic brain injury (TBI) is usually defined as a brain injury from an external force resulting in Glasgow Coma Scale of 3 to 8 (1) meaning that the patients are primarily unconscious or they gradually became unconscious after the injury. Severe TBI is associated with high mortality (2). About 30% of patients with severe TBI will die and 50% will suffer from at least moderate disability after 1 year, although some may show excellent recovery (3, 4). The initial severity assessment may be misleading due to frequently occurring confounders (prehospital sedation, hypoxia, inebriation, etc.), and the severity grading may change during the acute injury period, as TBI is a dynamic process with complex and heterogeneous pathophysiology. Early outcome prediction is challenging also due to impending secondary insults.

The primary brain injury results in a complex series of events, which generate a secondary brain injury process. Different insults belonging to a secondary brain injury are aggravated by impairment of energy metabolism that is a consequence of multiple potential mechanisms including both hypoxia and diffusion hypoxia (5, 6), increased non-oxidative processes (7), mitochondrial failure (8), and increased energy needs due to systemic trauma responses, seizures, or spreading depolarization (9, 10). The condition is further convoluted by heterogeneous temporal evolution of brain injury and individual differences between the patients (11, 12). The degree of disturbance of brain metabolism after TBI is also affected by treatment interventions, which are reflected in clinical patient outcome. Although the sustained metabolic crisis in the brain is mostly unsolvable by neurotrauma resuscitation and rigorous intracranial pressure (ICP) control (13–15), the monitoring of secondary brain injury events provides insight into early physiological insults experienced by the brain. It also provides an opportunity to treat physiological disturbances and predict the later pathophysiological course of TBI.

Sedation and neuromuscular blockade in the neurocritical care setting limit the ability of clinicians to obtain a reliable neurologic examination. Additionally, clinically observed deterioration often occurs as a late manifestation of secondary brain injury process. Multimodal neuromonitoring helps to detect, and in many circumstances to treat, cerebral ischemia. By monitoring invasively ICP, brain tissue oxygenation (PbtO2), cerebral perfusion autoregulation with the pressure reactivity index (PRx), and continuous electroencephalography, it is possible to assess and follow the gross physiology within the brain after severe TBI (16). Cerebral microdialysis provides a window into the underlying cellular metabolism of injured neurons by assessing the lactate/pyruvate ratio. An increase in this ratio reflects a decrease in the oxidative mitochondrial metabolism and mitochondrial failure. Thus, the basis for obtaining tissue metabolic data rests on the need for detecting a transition from aerobic to anaerobic metabolism. The switch to anaerobic metabolism is associated with poor neurological outcome in patients with TBI (9, 17, 18).

Cerebral microdialysis can be regarded as a type of local targeted metabolomics study, but there are also other means of assessing a vast spectrum of endogenous compounds with small molecular mass that serve as substrates and intermediates of biochemical pathways in the human body. In the continuing expansion of “omics” in biomedical research, the global study of metabolism at the molecular level, metabolomics, has enabled simultaneous determination of thousands of small molecules at various levels of cellular function due to the advances in systems biology. There is an on-going paradigm shift toward knowledge-based systemic “omics” studies leading to comprehensive metabolite profiling and fingerprint diagnostics in contrast to current hypothesis-driven research (19, 20).

Due to challenges in acute diagnostics, stratification and monitoring of treatment effects of severe TBI, several different methodologies to help the clinician have been studied, including different imaging modalities (13, 21–24), multimodal monitoring of brain and body physiology (9, 16, 25), and different protein biomarkers (26–30). Our current tools give little direct information about the brain’s wellbeing, not to mention predicting secondary injuries. Brain-specific or brain-enriched protein biomarkers have been expected to solve these problems, but the vast heterogeneity of TBIs, the variable damage of the blood–brain barrier (BBB), and problems in specificity have prevented them from reaching clinical use, although several studies have shown correlations with outcome (31–35). In this review, we will focus on severe TBI, because its vast complexity poses a special challenge for diagnostics. We discuss the results and prospects of metabolomics to overcome these challenges, as this methodology may be able to offer individual fingerprint characterization of the on-going pathophysiological events, without many problems that face the use of proteins as brain biomarkers.

## Metabolomics

### Clinical Need for New Blood-Based Biomarkers of Severe TBI

Traumatic brain injury has been a clinically challenging problem for several reasons, including poorly understood complex pathophysiology that behaves unpredictably and vast patient and injury heterogeneity. There are a number of sensitive organ-based biomarkers in clinical use for medical emergencies (36) and oncology diagnostics (37). Accordingly, similar markers for TBI have been searched for, in order to assess the nature and severity of the injury and patient outcome (38, 39).

For an ideal universal molecular biomarker of TBI, the compound should be readily measurable in peripheral venous blood or non-invasively collected biological fluid, as diagnostics from cerebrospinal fluid or cerebral microdialyzates are too invasive methods in evaluating mild or moderate TBI. Moreover, severe TBI sets special requirements for biomarkers. For a biomarker to be useful in severe TBI cases, it needs to show changes during the initial stages such as transition to mild cerebral energy crisis or regional swelling. The changes need to be detected prior to the onset of global cerebral energy failure and uncontrollable ICP elevation. Depending on the nature of the diagnostic aim, a biomarker of TBI should be able to confirm the presence or absence of TBI, assess the severity and nature of TBI, monitor treatment effects and predict outcome. Furthermore, validation of a biomarker needs to be linked to established clinically relevant indicators of disease severity, e.g., Glasgow coma scale (1), acute imaging findings (such as acute head computed tomography or magnetic resonance imaging), brain tissue fate as assessed with different methods (40–42), or outcome (43).

It appears highly unlikely that a single biomarker could accurately describe these different clinical needs in a case of severe TBI at the emergency department and intensive care unit. This is because patients and injuries are highly heterogeneous and there is significant uncontrolled variability even within the same category of TBI severity, merely as assessed by rough clinical measures. In the case of an extremely complex disease, such as severe TBI, the inherent variability needs to be taken into account, because the molecular biomarkers might not be fundamentally related to TBI but rather to normal and reactive physiological processes and protective responses, such as those related to age, gender, diet, CNS comorbidities, and extracranial injuries. Therefore, instead of measuring a single TBI-sensitive biomarker, there is a need for comprehensive injury-sensitive biochemical profiling and individual fingerprint diagnostics.

### Challenges in TBI Biomarker Research

Protein-based biomarkers have partially failed to fill the expectations in diagnostics of TBI. The problems have been one-dimensional diagnostic perspectives, sensitivity and specificity for TBI and brain, and the inability to pass an intact BBB. The variability in dysfunction of the BBB as a result of TBI strongly affects the performance of proteins to serve as reliable biomarkers of intracranial events. Small molecules with molecular mass under 1,000 Da are more readily able to pass an intact BBB and are thus much more independent from fluctuating and immeasurable confounders related to BBB dysfunction, which is one of the cornerstones of TBI pathophysiology (44). As metabolic profiling can detect and measure a large number of substances, it may enable accurate characterization and stratification of the TBIs for targeted therapies. Blood-based metabolomics profiling is the preferred method due to practical reasons. Although better brain specificity could be achieved by employing CSF analytics, there is no clinical justification to use CSF for biomarker assessment in cases where it is not necessary.

Intracranial dynamics is efficiently monitored by the current methods such as invasive monitoring of ICP, PRx, and PbtO_2_, reflecting global and regional changes in patients with severe TBI. The validated methods for monitoring metabolic crisis in the brain following severe TBI have been brain microdialysis, arterio-jugular venous differences, and positron emission tomography, of which the first-mentioned is not universally available and the latter is neither universally available nor suitable for patients with unstable or intractable ICP.

### Metabolomics As an Opportunity for TBI Diagnostics

Metabolomics is a global approach to study the structure, function, and interactions of low molecular weight metabolites in cells, tissues, and biofluids (45). Unlike in the setting of protein diagnostics, the metabolic profile is a snapshot that provides a window into the *in vivo* enzymatic activity of the brain and body, because free metabolite concentrations affect, and are affected by the global metabolic activity (46, 47). Metabolites can be studied and compared with physiological and pathophysiological conditions, allowing better and more comprehensive understanding of disease processes.

As simultaneous determination of a plethora of molecules has become possible due to the new analytical technologies in systems biology, metabolomics enables a conception of biological organism as a network of interacting cells and their metabolites. Given the highly complex nature of the human brain, metabolomics can be utilized to address the biomolecular interaction networks of the brain in health and disease (20, 48).

### Technology and Statistical Methods Used in Metabolomics Diagnostics

Several techniques for metabolomics have been applied in discovery and analysis of different biomarkers. Most methods are based on mass spectrometry (MS), typically combined with chromatographic separation techniques, such as gas or liquid chromatography (GC or LC). Proton nuclear magnetic resonance (^1^H-NMR) has also been widely applied. The advantage of NMR over MS-based methods is the relative simplicity of the sample preparation required. Additionally, ^1^H-NMR suffers less from batch-to-batch variation observed in global MS-based approaches. However, because of its poorer sensitivity (micromolar concentration range), it is not so useful for biomarkers of TBI, which are typically present at lower levels (picomolar to nanomolar) in the serum. Another key limitation to ^1^H-NMR is the resolution of the resultant spectra. Typically, metabolomic profiling occurs on high field systems (600 MHz and above), but there is still significant overlap of the peaks (49). This leads to problems with metabolite identification, even if two-dimensional experiments are performed (50). Furthermore, it makes interpreting the increase in NMR signals difficult as it is often unclear, which metabolite causes the increase in signal if multiple metabolites overlap. On the other hand, MS-based methods have the advantage of being more sensitive than NMR (picomolar-micromolar), which allows for the greater detection of metabolites. This higher number of metabolites allows for the greater coverage of biochemical pathways allowing the mechanisms by which the metabolites are changing to be understood. However, one drawback to MS-based methods is the sample preparation, which is typically based on extraction or protein precipitation, which can crate a source of variation into the analysis. This variation can be corrected by using appropriate class-based standards during the extraction or protein precipitation.

In metabolomics, there are basically two types of methodologies used, namely untargeted and targeted analyses. Untargeted analyses are typically applied in biomarker discovery studies, and they correspondingly aim at analyzing comprehensive metabolic profiles. These methods are usually semi-quantitative, i.e., relative concentrations of metabolites are determined between the study groups. The targeted analyses are generally quantitative, and they are limited to the analysis of specific target metabolites. This is because to fully quantify a metabolite, a standard curve of known concentrations needs to be generated. When profiling all metabolites in a biofluid, it is not possible to have a complete set of pure compounds to generate these standard curves. Therefore, class-based internal standards are used, which allow a relative concentration to be calculated. This is known as semi-quantification. In the more targeted approach where the number of metabolites being analyzed is smaller, it is possible to generate standard curves for all metabolites. An appropriate internal standard to correct for matrix effects during the run can then be used. Matrix effects occur when multiple metabolites elute from the column at the same time causing ion suppression in the MS (51). Therefore, depending on the sample and its preparation, it is possible to get an apparent reduction in the specific metabolite concentration, which prevents absolute quantitation (52). To minimize these matrix effects requires, the presence of a heavy isotope standard, which elutes at the same time as the metabolite being studied (53). These heavy isotope standards are not always available for the whole metabolome and if they were available, it may be prohibitively expensive.

In an untargeted approach, it is still not possible to analyze the whole metabolome with a single method, because of the large diversity of the metabolites, both in terms of chemical diversity and concentration range. First of all, it is not possible to extract both hydrophilic and hydrophobic metabolites with a single method in a robust manner. In untargeted analysis, the most common approach is to use either GC or LC combined with MS. GC-based systems are suitable for volatile and semi-volatile metabolites, while LC can in principle be used for all types of metabolites. The advantages of GC-based systems are good separation efficiency, capability of analysis of very polar and semi-polar analytes in a single method, and the availability of large commercial mass spectral libraries for the identification of metabolites. The main disadvantage of the GC-based methods is the unsuitability for non-volatile metabolites, and that derivatization is needed for the analysis of polar compounds. The main advantage of the LC–MS methods, on the other hand, is the simpler sample preparation, and the applicability to a wider range of metabolites. However, the LC–MS suffers from matrix effects, which make the quantification more challenging in untargeted methods as compared to GC-based methods. With LC, high-resolution accurate MS systems capable of tandem mass measurements have been most commonly applied for untargeted metabolomic analyses, particularly using quadrupole time-of-flight MS (QTOFMS) and Orbitrap MS systems. With GC, TOFMS and QTOFMS systems are also widely applied for metabolomics, although the simple quadruple MS systems are still the most commonly employed method.

**Figure 1:**
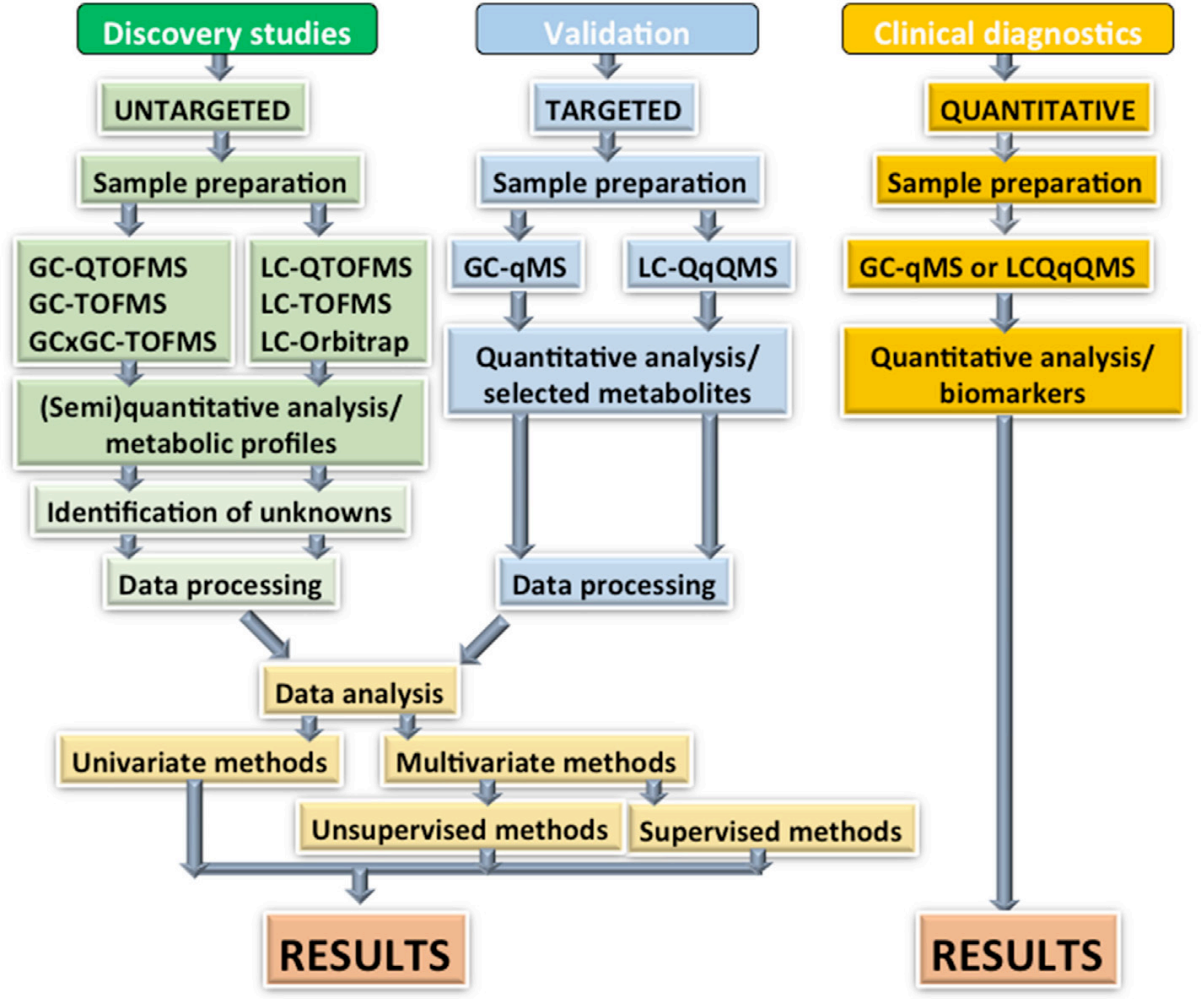
Overview of analytical strategies for metabolomics: there are three key phases required for the development of metabolomics biomarkers if they are to be used in a clinical setting. The initial discovery phase can capture a large number of metabolites using a combination of GC and LC-based techniques. High separation efficiency preferably combined with high mass accuracy is important at this stage due to the high number of metabolites with similar masses. These techniques generate semi-quantitative measurements, which makes them unsuitable for clinical practice. These techniques can also identify unknown compounds, which potentially require time and extra experiments to identify. The validation stage is a key to any biomarker study. Once the metabolites of interest have been identified from the discovery phase, a more targeted method must be developed aimed at only quantifying these. This method needs to be applied to the original data as well as new independent samples to avoid overmodeling. Finally, a fully quantitative assay must be developed for use in the clinic. The analysis needs to be streamlined and user friendly to ensure cost efficiency. Key: GC, gas chromatography; GCxGC, two-dimensional gas chromatography; LC, liquid chromatography; Q, single quadrupole; QqQ, triple quadrupole; TOF, time-of-flight mass spectrometry.

In untargeted metabolomics approaches, raw MS data first need to be processed before it can be analyzed by statistical approaches. Several open source software packages have been developed for this purpose, and MS vendors currently offer their own solutions for metabolomics data processing. Among the open source tools, MZmine (54) and XCMS (55) have been the most commonly applied for LC–MS based approaches. Once the data processing step is complete and the data is made available, e.g., in the matrix format, the metabolic profiles can be studied by a variety of statistical approaches, depending on the experimental setting. The general statistical considerations for metabolomics (56) or any other high-dimensional “omics” data (57) need to be applied. The univariate and multivariate methods applicable to metabolomics/lipidomics data analysis have been reviewed extensively (58). However, to summarize briefly, the use of both univariate and multivariate techniques are required depending on the questions raised in the study. One of the largest problems in metabolomics or lipids is the great number of possible species identified by the analytical technique used. This results in high-dimensional data leading to obscure plots (58). To overcome this issue, multivariate techniques such as principal component analysis are used to group the data. For lipidomic data, clustering techniques can be especially powerful due to the relatively small number of lipid classes (approximately 10) that can be measured in untargeted approaches. Lipids in these classes tend to correlate with each other and change consistently in disease (58). The univariate statistical methods can be very useful in visualizing the data. Heatmaps showing the correlation of metabolites or lipids are often used to show species, which are co-regulated. Box and whisker plots coupled to p-value analysis are used to filter and visualize the sample-to-sample variation within a metabolite of interest. One common pitfall when applying predictive modeling (e.g., for biomarker discovery) from multivariate data is the lack of proper validation. In the literature, the most commonly applied approach for this purpose is the partial least squares discriminant analysis (PLS-DA) (59). However, this approach suffers from so-called overfitting, and the reported models, if developed and tested on the same dataset, tend to be over-optimistic, particularly if improper internal validation is applied. Ideally, the PLS-DA model (or those derived from other multivariate methods) needs to be tested and reported on an independent dataset, which has not been used for the model development. A typical workflow of a metabolomics study is demonstrated in Figure 1.

### Metabolomics Applied to Diagnostics of TBI

There are three studies that have utilized a lipidomics approach in TBI research: in a murine model (60) and in humans (61, 62), while two studies have employed metabolomics approach in humans with TBI (63, 64). Additionally, there are ^1^H-NMR metabolomics studies conducted on murine brain tissue specimens and plasma (65), and on human CSF (66). The metabolites that have been significantly associated with TBI in these studies are listed in Table 1.

**Table 1:** Metabolites that have been reported to be significantly associated with traumatic brain injury (TBI).

Metabolite name	Metabolite type	Source	Quantity in TBI	Reference
Decanoic acid	Medium-chain fatty acid	Human serum	Upregulated	(64)
Octanoic acid	Medium-chain fatty acid	Human serum	Upregulated	(64)
2,3-bisphosphoglyceric acid	Glyceric acid derivative	Human serum	Upregulated	(64)
Alanine	Amino acid	Human serum	Downregulated	(64)
Serine	Amino acid	Human serum	Downregulated	(64)
Indole-3-propionic acid	Tryptophan deamination product	Human serum	Downregulated	(64)
12 different choline plasmalogens	Glycerophospholipids	Human plasma	N/A	(63)
Acylcarnitine C5	Amino acid	Human plasma	N/A	(63)
Putrescine	Polyamine	Human plasma	N/A	(63)
Formate	Anion	Human plasma	N/A	(63)
Methanol	Alcohol	Human plasma	N/A	(63)
Succinate	Dicarboxylic acid	Human plasma	N/A	(63)
Propylene glycol	Alcohol	Human CSF	Upregulated	(66)
Creatinine	Imidazoline derivative	Human CSF	Downregulated	(46)
Ascorbate	Salt of ascorbic acid	Rat brain	Downregulated	(65)
Glutamate	Amino acid	Rat brain	Downregulated	(65)
Phosphocholine	Choline derivative	Rat brain	Downregulated	(65)
Glycerophosphocholine	Choline derivative	Rat brain	Downregulated	(65)
N-acetylaspartate	Derivative of aspartic acid	Rat brain	Downregulated	(65)

Viant and colleagues studied brain tissue specimens and plasma of rats that were exposed to fluid percussion injury (65). The samples were obtained 1 h after injury. They found decreased levels of ascorbate in cortex and hippocampus, glutamate in the cortex and hippocampus, phosphocholine and glycerophosphocholine in the cortex and hippocampus, and N-acetylaspartate in the cortex and hippocampus. While TBI had an effect on the metabolomic profile found in brain tissue, no clear effects were detected in plasma samples (65).

Glenn and colleagues studied single CSF samples of 44 patients with severe TBI and 13 non-injured control patients with normal pressure hydrocephalus or unruptured intracranial aneurysms (66). The ^1^H-NMR spectra showed prominent peaks for lactate, acetate, beta-glucose, total creatine, pyruvate, glutamine, alanine, creatinine, alpha-glucose, and a doublet propylene glycol. Patients with severe TBI had significantly higher levels of propylene glycol and lower levels of creatinine than the controls. The timing of CSF draws were not matched with clinical events, but in multivariate model, the profile including propylene glycol, glutamine, alpha-glucose, and creatinine was associated with cerebral metabolic rate of oxygen, ICP, and outcome. The study did not provide any information on statistical values (66).

Daley and colleagues reported that in adolescent male hockey players who had sustained a concussion, a set of metabolites relying notably on glycerophospholipids accounted for 82% of the variance between 12 concussed and 17 non-concussed athletes. The group utilized ^1^H-NMR and a method using both direct injection and LC combined with tandem MS. The two methods together cover amino acids, acyl carnitines, specific lipids, and some amines (FIA/LC-MS) as well as glucose, specific hydroxyl acids, and ketone bodies (^1^H-NMR). The method combining multivariate statistical analysis and machine learning exhibited 92% accuracy rate in diagnosing a concussion (63). However, the possible cofounding factors, such as diet, time from last meal or BMI were not accounted for in the statistical analyses, and the results have not been independently validated in another study group.

TBIcare investigators and Turku Centre for Biotechnology Systems Medicine research group applied comprehensive metabolic profiling of serum samples from two large independent cohorts of patients with full spectrum of TBI and orthopedic injuries (64). Serum metabolomic profiles from 144 patients with mild, moderate or severe TBI were investigated. A control group comprised 28 patients with acute orthopedic injuries without an acute or earlier TBI. The samples were taken upon admission to emergency department (<12 h after the injury). Two-dimensional GC coupled to time-of-flight MS was utilized to analyze the serum samples. The metabolite profiles of the four patient groups were compared to an independent validation cohort from Addenbrooke’s Hospital (Cambridge, UK) comprising 67 patients with TBIs of all severities and patients with orthopedic injuries. Decanoic and octanoic acid, which are medium-chain fatty acids, and sugar derivatives including 2,3-bisphosphoglyceric acid were strongly associated with the severity of TBI. These metabolites were detected in significantly higher concentrations in patients with TBI (with or without other injuries) than in patients with orthopedic injuries without any suspicion of CNS trauma. Metabolite levels in patients with mild TBI followed the same pattern as in more severe TBI, but the magnitude of change compared to controls was less than in severe TBI. Brain microdialyzates were also analyzed from 12 samples acquired from patients with severe TBI in the validation cohort, in order to compare the significant serum metabolites with brain extracellular metabolites. The levels of top-ranking serum metabolites associated with TBI correlated highly with their levels in brain microdialyzates, thus suggesting disruption of the BBB. As a second main aim of the study, a prognostic model was developed to discriminate patients with favorable (Glasgow Outcome Scale extended 5–8) and unfavorable (Glasgow Outcome Scale extended 1–4) outcome. In the discovery cohort, the performance of the model reached an area under curve (AUC) of 0.90 (95% CI 0.83–0.95) and in validation cohort an AUC of 0.84 (95% CI 0.75–0.89) (Table 2). The added value of the prognostic model was studied together with the established CRASH prognostic model (67), consisting of clinical variables. The stand-alone AUC of the CRASH model was 0.74 in the validation cohort. When the top-ranking metabolites (decanoic acid and pentitol-3-desoxy) from prognostic metabolomic model were combined to CRASH model, AUC reached 0.80. The results demonstrate that TBI is associated with a specific metabolic profile, which is exacerbated proportionally to the severity of TBI.

**Table 2:** Serum metabolites of which levels distinguish between patients with favorable and unfavorable outcome upon admission in a in two independent cohorts of patients (*n* = 144 and *n* = 67, respectively) with full spectrum of traumatic brain injury (64).

Metabolite name	Description
Decanoic acid	Medium-chain fatty acid
Octanoic acid	Medium-chain fatty acid
Tryptophan	Alpha-amino acid
Butanal, 2,3,4-trishydroxy-3-methoxy	Sugar derivative
3-Oxobutanoic acid	Beta-keto acid

*In addition to the above metabolites, four unknown sugar derivatives, a phenolic metabolite and an unknown amino acid form a model that predicts patient outcomes with AUC of 0.84 (64)*.

## Concluding Remarks

A new era is emerging in the diagnostics of TBI. There is a paradigm shift toward comprehensive “omics” studies leading to proteomics and metabolomics profiling and fingerprint diagnostics, in contrast to current clinical diagnostics with non-specific and unreliable clinical markers. In the future, a confluence of multi-time-point proteomics and metabolomics diagnostics and advanced imaging studies will highly likely offer more precise stratification and outcome prediction, while individual point-of-care biomarker monitoring of the injured brain will provide means for assessment of intervention efficacy.

At the moment, the number of papers on metabolomics in TBI is small. The identified metabolites associated with TBI are diverse and they have arises from studies that have employed variable methods. Human TBI is in many ways different from experimental TBI models that produce standardized injuries, which never occur in humans. Animal models could be used to evaluate the origin of some metabolites, but otherwise there is no reason to expect why reproducibility between species would be better for metabolomics compared to, e.g., protein biomarkers or pharmacological interventions in TBI, which have given disappointing results from bench to bedside.

The search for clinically relevant TBI fingerprints has just begun. The identified human serum “TBI metabotype” offers a new avenue for the development of next generation diagnostic, prognostic, monitoring and surrogate markers of broad spectrum of TBIs. At the moment, it is impossible to state that this metabotype is brain-specific, but the current results show that the key metabolites are significantly upregulated in patients with TBI as compared to orthopedic controls.

Compared to earlier pursuits in finding a brain- and TBI-specific single compound in blood, the value of metabolomics is in partly avoiding limitations arising from BBB permeability. Metabolomics provides a fingerprint profile of multiple processes, which is important in complex diseases such as TBI. The next steps require explorative studies for different types of injuries at different points in time and correlating the results with clinical and imaging parameters. These tasks will strongly rely on systems medicine approaches and artificial intelligence to interpret the results in different clinical settings. The ultimate challenge lies in validating future metabolite panels for different clinical needs and at variable time points in this vastly heterogeneous patient population.

## Author Contributions

All authors devised the review article. JP and AD drafted the first versions of the paper with critical contributions from MO, TH, and OT. All authors reviewed, edited, and approved the final version.

## Conflict of Interest Statement

The authors declare that the research was conducted in the absence of any commercial or financial relationships that could be construed as a potential conflict of interest.

## References

[1] Teasdale G Jennett B Assessment of coma and impaired consciousness. A practical scale. Lancet (1974) 2:81–4. 10.1016/S0140-6736(74)91639-0 4136544

[2] Brain Trauma Foundation, American Association of Neurological Surgeons, Congress of Neurological Surgeons, Joint Section on Neurotrauma and Critical Care, AANS/CNS Carney NA Ghajar J Guidelines for the management of severe traumatic brain injury. Introduction. J Neurotrauma (2007) 24(Suppl 1):S1–2. 10.1089/neu.2007.9997 17511535

[3] Thornhill S Teasdale GM Murray GD McEwen J Roy CW Penny KI. Disability in young people and adults one year after head injury: prospective cohort study. BMJ (2000) 320:1631–5. 10.1136/bmj.320.7250.1631 10856063 PMC27407

[4] Myburgh JA Cooper DJ Finfer SR Venkatesh B Jones D Higgins A Epidemiology and 12-month outcomes from traumatic brain injury in Australia and New Zealand. J Trauma (2008) 64:854–62. 10.1097/TA.0b013e3180340e77 18404048

[5] Coles J Fryer T Smielewski P Chatfield D Steiner L Johnston A Incidence and mechanisms of cerebral ischemia in early clinical head injury. J Cereb Blood Flow Metab (2004) 24:202–11. 10.1097/01.WCB.0000103022.98348.24 14747747

[6] Menon D Coles J Gupta A Fryer T Smielewski P Chatfield D Diffusion limited oxygen delivery following head injury. Crit Care Med (2004) 32:1384–90. 10.1097/01.CCM.0000127777.16609.08 15187523

[7] Kawamata T Katayama Y Hovda DA Yoshino A Becker DP. Lactate accumulation following concussive brain injury: the role of ionic fluxes induced by excitatory amino acids. Brain Res (1995) 674:196–204. 10.1016/0006-8993(94)01444-M 7540925

[8] Lakshmanan R Loo JA Drake T Leblanc J Ytterberg AJ McArthur DL Metabolic crisis after traumatic brain injury is associated with a novel microdialysis proteome. Neurocrit Care (2010) 12:324–36. 10.1007/s12028-010-9342-5 20225002 PMC4347948

[9] Timofeev I Carpenter KLH Nortje J Al Rawi P O’Connell M Czosnyka M Cerebral extracellular chemistry and outcome following traumatic brain injury: a microdialysis study of 223 patients. Brain (2011) 134:484–94. 10.1093/brain/awq353 21247930

[10] Dienel GA Rothman DL Nordstrom CH Microdialysate concentration changes do not provide sufficient information to evaluate metabolic effects of lactate supplementation in brain-injured patients. J Cereb Blood Flow Metab (2016) 36:1844–64. 10.1177/0271678X16666552 27604313 PMC5094313

[11] Chesnut RM Marshall LF Klauber MR Blunt BA Baldwin N Eisenberg HM The role of secondary brain injury in determining outcome from severe head injury. J Trauma (1993) 34:216–22. 10.1097/00005373-199302000-00006 8459458

[12] Manley G Knudson MM Morabito D Damron S Erickson V Pitts L. Hypotension, hypoxia, and head injury: frequency, duration, and consequences. Arch Surg (2001) 136:1118–23. 10.1001/archsurg.136.10.1118 11585502

[13] Vespa P Bergsneider M Hattori N Wu H Huang S Martin N Metabolic crisis without brain ischemia is common after traumatic brain injury: a combined microdialysis and positron emission tomography study. J Cereb Blood Flow Metab (2005) 25:763–74. 10.1038/sj.jcbfm.9600073 15716852 PMC4347944

[14] Stein N McArthur D Etchepare M Vespa P. Early cerebral metabolic crisis after TBI influences outcome despite adequate hemodynamic resuscitation. Neurocrit Care (2012) 17:49–57. 10.1007/s12028-012-9708-y 22528283

[15] Chesnut RM Temkin N Carney N Dikmen S Rondina C Videtta W A trial of intracranial-pressure monitoring in traumatic brain injury. N Engl J Med (2012) 367:2471–81. 10.1056/NEJMoa1207363 23234472 PMC3565432

[16] Lazaridis C Robertson C The role of multimodal invasive monitoring in acute traumatic brain injury. Neurosurg Clin N Am (2016) 27:509–17. 10.1016/j.nec.2016.05.010 27637400

[17] Bellander BM Cantais E Enblad P Hutchinson P Nordstrom CH Robertson C Consensus meeting on microdialysis in neurointensive care. Intensive Care Med (2004) 30:2166–9. 10.1007/s00134-004-2461-8 15549254

[18] Carpenter KL Czosnyka M Jalloh I Newcombe VF Helmy A Shannon RJ Systemic, local, and imaging biomarkers of brain injury: more needed, and better use of those already established? Front Neurol (2015) 6:26. 10.3389/fneur.2015.00026 25741315 PMC4332345

[19] Lichtman JW Pfister H Shavit N. The big data challenges of connectomics. Nat Neurosci (2014) 17:1448–54. 10.1038/nn.3837 25349911 PMC4412267

[20] Vasilopoulou CG Margarity M Klapa MI. Metabolomic analysis in brain research: opportunities and challenges. Front Physiol (2016) 7:183. 10.3389/fphys.2016.00183 27252656 PMC4878281

[21] Marshall LF Marshall SB Klauber MR van Berkum Clark M Eisenberg HM Jane JA A new classification of head injury based on computerized tomography. J Neurosurg (1991) 75:S14–20.

[22] Maas AI Hukkelhoven CW Marshall LF Steyerberg EW. Prediction of outcome in traumatic brain injury with computed tomographic characteristics: a comparison between the computed tomographic classification and combinations of computed tomographic predictors. Neurosurgery (2005) 57:1173,82. 10.1227/01.NEU.0000186013.63046.6B 16331165

[23] Sidaros A Engberg A Sidaros K Liptrot M Herning M Petersen P Diffusion tensor imaging during recovery from severe traumatic brain injury and relation to clinical outcome: a longitudinal study. Brain (2008) 131:559–72. 10.1093/brain/awm294 18083753

[24] Raj R Siironen J Skrifvars MB Hernesniemi J Kivisaari R Predicting outcome in traumatic brain injury: development of a novel computerized tomography classification system (Helsinki computerized tomography score). Neurosurgery (2014) 75:632,46 10.1227/NEU.0000000000000533 25181434

[25] Sharma D Vavilala M. Perioperative management of adult traumatic brain injury. Anesthesiol Clin (2012) 30:333–46. 10.1016/j.anclin.2012.04.003 22901613 PMC3424485

[26] Thelin E Nelson D Bellander B. Secondary peaks of S100B in serum relate to subsequent radiological pathology in traumatic brain injury. Neurocrit Care (2014) 20:217–29. 10.1007/s12028-013-9916-0 24146416

[27] Al Nimer F Thelin E Nyström H Dring A Svenningsson A Piehl F Comparative assessment of the prognostic value of biomarkers in traumatic brain injury reveals an independent role for serum levels of neurofilament light. PLoS One (2015) 10:e0132177. 10.1371/journal.pone.0132177 26136237 PMC4489843

[28] Papa L Brophy G Welch R Lewis L Braga C Tan C Time course and diagnostic accuracy of glial and neuronal blood biomarkers GFAP and UCH-L1 in a large cohort of trauma patients with and without mild traumatic brain injury. JAMA Neurol (2016) 73:551–60. 10.1001/jamaneurol.2016.0039 27018834 PMC8805143

[29] Posti JP Takala RS Runtti H Newcombe VF Outtrim J Katila AJ The levels of glial fibrillary acidic protein and ubiquitin C-terminal hydrolase-L1 during the first week after a traumatic brain injury: correlations with clinical and imaging findings. Neurosurgery (2016) 79:456–64. 10.1227/NEU.0000000000001226 26963330

[30] Thelin E Jeppsson E Frostell A Svensson M Mondello S Bellander B Utility of neuron-specific enolase in traumatic brain injury; relations to S100B levels, outcome, and extracranial injury severity. Crit Care (2016) 20:285. 10.1186/s13054-016-1450-y 27604350 PMC5015335

[31] Mondello S Papa L Buki A Bullock MR Czeiter E Tortella FC Neuronal and glial markers are differently associated with computed tomography findings and outcome in patients with severe traumatic brain injury: a case control study. Crit Care (2011) 15:R156. 10.1186/cc10286 21702960 PMC3219030

[32] Czeiter E Mondello S Kovacs N Sandor J Gabrielli A Schmid K Brain injury biomarkers may improve the predictive power of the IMPACT outcome calculator. J Neurotrauma (2012) 29:1770–8. 10.1089/neu.2011.2127 22435839 PMC3409455

[33] Thelin E Johannesson L Nelson D Bellander B S100B is an important outcome predictor in traumatic brain injury. J Neurotrauma (2013) 30:519–28. 10.1089/neu.2012.2553 23297751

[34] Takala RS Posti JP Runtti H Newcombe VF Outtrim J Katila AJ GFAP and UCH-L1 as outcome predictors in traumatic brain injury. World Neurosurg (2016) 87:8–20. 10.1016/j.wneu.2015.10.066 26547005

[35] Papa L Silvestri S Brophy GM Giordano P Falk JL Braga CF GFAP out-performs S100beta in detecting traumatic intracranial lesions on computed tomography in trauma patients with mild traumatic brain injury and those with extracranial lesions. J Neurotrauma (2014) 31:1815–22. 10.1089/neu.2013.3245 24903744 PMC4224051

[36] Long B Koyfman A. Ready for prime time? Biomarkers in sepsis. Emerg Med Clin North Am (2017) 35:109–22. 10.1016/j.emc.2016.09.004 27908327

[37] Holdenrieder S Pagliaro L Morgenstern D Dayyani F Clinically meaningful use of blood tumor markers in oncology. Biomed Res Int (2016) 2016:9795269 10.1155/2016/9795269 28042579 PMC5155072

[38] Bakay RA Sweeney KM Wood JH. Pathophysiology of cerebrospinal fluid in head injury: part 2. Biochemical markers for central nervous system trauma. Neurosurgery (1986) 18:376–82. 10.1097/00006123-198603000-00026 3010171

[39] Zetterberg H Smith DH Blennow K. Biomarkers of mild traumatic brain injury in cerebrospinal fluid and blood. Nat Rev Neurol (2013) 9:201–10. 10.1038/nrneurol.2013.9 23399646 PMC4513656

[40] Ledig C Heckemann R Hammers A Lopez J Newcombe VFJ Makropoulos A Robust whole-brain segmentation: application to traumatic brain injury. Med Image Anal (2015) 21:40–58. 10.1016/j.media.2014.12.003 25596765

[41] Newcombe VFJ Correia M Ledig C Abate M Outtrim J Chatfield D Dynamic changes in white matter abnormalities correlate with late improvement and deterioration following TBI: a diffusion tensor imaging study. Neurorehabil Neural Repair (2016) 30:49–62. 10.1177/1545968315584004 25921349

[42] Mohammadian M Roine T Hirvonen J Kurki T Ala Seppälä H Frantzén J High angular resolution diffusion-weighted imaging in mild traumatic brain injury. Neuroimage Clin (2017) 13:174–80. 10.1016/j.nicl.2016.11.016 27981032 PMC5144744

[43] Wilson JT Pettigrew LE Teasdale GM Structured interviews for the Glasgow Outcome Scale and the extended Glasgow Outcome Scale: guidelines for their use. J Neurotrauma (1998) 15:573–85. 10.1089/neu.1998.15.573 9726257

[44] Saw M Chamberlain J Barr M Morgan MPG Burnett J Ho K. Differential disruption of blood-brain barrier in severe traumatic brain injury. Neurocrit Care (2014) 20:209–16. 10.1007/s12028-013-9933-z 24233818

[45] Ma H Sorokin A Mazein A Selkov A Selkov E Demin O The Edinburgh human metabolic network reconstruction and its functional analysis. Mol Syst Biol (2007) 3:135 10.1038/msb4100177 17882155 PMC2013923

[46] Patti G Yanes O Siuzdak G. Innovation: metabolomics: the apogee of the omics trilogy. Nat Rev Mol Cell Biol (2012) 13:263–9. 10.1038/nrm3314 22436749 PMC3682684

[47] Hollywood K Brison D Goodacre R. Metabolomics: current technologies and future trends. Proteomics (2006) 6:4716–23. 10.1002/pmic.200600106 16888765

[48] Vidal M Cusick M Barabási A Interactome networks and human disease. Cell (2011) 144:986–98. 10.1016/j.cell.2011.02.016 21414488 PMC3102045

[49] Dickens A Larkin J Davis B Griffin J Claridge TDW Sibson N NMR-based metabolomics separates the distinct stages of disease in a chronic relapsing model of multiple sclerosis. J Neuroimmune Pharmacol (2015) 10:435–44. 10.1007/s11481-015-9622-0 26155956

[50] Dickens A Larkin J Griffin J Cavey A Matthews L Turner M A type 2 biomarker separates relapsing-remitting from secondary progressive multiple sclerosis. Neurology (2014) 83:1492–9. 10.1212/WNL.0000000000000905 25253748 PMC4222850

[51] King R Bonfiglio R Fernandez Metzler C Miller Stein C Olah T. Mechanistic investigation of ionization suppression in electrospray ionization. J Am Soc Mass Spectrom (2000) 11:942–50. 10.1016/S1044-0305(00)00163-X 11073257

[52] Dunn WB Ellis DI Metabolomics: current analytical platforms and methodologies. Trends Anal Chem (2005) 24:285–94. 10.1016/j.trac.2004.11.021

[53] Matuszewski BK Constanzer ML Chavez Eng CM. Strategies for the assessment of matrix effect in quantitative bioanalytical methods based on HPLC-MS/MS. Anal Chem (2003) 75:3019–30. 10.1021/ac020361s 12964746

[54] Pluskal T Castillo S Villar Briones A Oresic M. MZmine 2: modular framework for processing, visualizing, and analyzing mass spectrometry-based molecular profile data. BMC Bioinformatics (2010) 11:395. 10.1186/1471-2105-11-395 20650010 PMC2918584

[55] Gowda H Ivanisevic J Johnson C Kurczy M Benton HP Rinehart D Interactive XCMS Online: simplifying advanced metabolomic data processing and subsequent statistical analyses. Anal Chem (2014) 86:6931–9. 10.1021/ac500734c 24934772 PMC4215863

[56] Broadhurst D Kell D Statistical strategies for avoiding false discoveries in metabolomics and related experiments. Metabolomics (2016) 2:171 10.1007/s11306-006-0037-z

[57] Ransohoff D. Bias as a threat to the validity of cancer molecular-marker research. Nat Rev Cancer (2005) 5:142–9. 10.1038/nrc1550 15685197

[58] Niemelä P Castillo S Sysi Aho M Oresic M. Bioinformatics and computational methods for lipidomics. J Chromatogr B Analyt Technol Biomed Life Sci (2009) 877:2855–62. 10.1016/j.jchromb.2009.01.025 19200789

[59] Boulesteix A Strimmer K. Partial least squares: a versatile tool for the analysis of high-dimensional genomic data. Brief Bioinform (2007) 8:32–44. 10.1093/bib/bbl016 16772269

[60] Sheth S Iavarone A Liebeskind D Won S Swanson R. Targeted lipid profiling discovers plasma biomarkers of acute brain injury. PLoS One (2015) 10:e0129735. 10.1371/journal.pone.0129735 26076478 PMC4468135

[61] Emmerich T Abdullah L Crynen G Dretsch M Evans J Ait Ghezala G Plasma lipidomic profiling in a military population of mild traumatic brain injury and post-traumatic stress disorder with apolipoprotein E ɛ4-dependent effect. J Neurotrauma (2016) 33:1331–48. 10.1089/neu.2015.4061 26714394

[62] Emmerich T Abdullah L Ojo J Mouzon B Nguyen T Laco G Mild TBI results in a long-term decrease in circulating phospholipids in a mouse model of injury. Neuromolecular Med (2017) 19:122–35. 10.1007/s12017-016-8436-4 27540748

[63] Daley M Dekaban G Bartha R Brown A Stewart TC Doherty T Metabolomics profiling of concussion in adolescent male hockey players: a novel diagnostic method. Metabolomics (2016) 12:184 10.1007/s11306-016-1131-5

[64] Oresic M Posti JP Kamstrup-Nielsen MH Takala RS Lingsma HF Mattila I Human serum metabolites associate with severity and patient outcomes in traumatic brain injury. EBioMedicine (2016) 12:118–26. 10.1016/j.ebiom.2016.07.015 27665050 PMC5078571

[65] Viant M Lyeth B Miller M Berman R. An NMR metabolomic investigation of early metabolic disturbances following traumatic brain injury in a mammalian model. NMR Biomed (2005) 18:507–16. 10.1002/nbm.980 16177961

[66] Glenn T Hirt D Mendez G McArthur D Sturtevant R Wolahan S Metabolomic analysis of cerebral spinal fluid from patients with severe brain injury. Acta Neurochir Suppl (2013) 118:115–9. 10.1007/978-3-7091-1434-6_20 23564115

[67] MRC CRASH Trial Collaborators Perel P Arango M Clayton T Edwards P Komolafe E Predicting outcome after traumatic brain injury: practical prognostic models based on large cohort of international patients. BMJ (2008) 336:425–9. 10.1136/bmj.39461.643438.25 18270239 PMC2249681

